# The Congress Impact Factor: A proposal from board members of the World Society of Emergency Surgeons.it (WSES) and Academy of Emergency Medicine and Care (AcEMC)

**DOI:** 10.12688/f1000research.15429.2

**Published:** 2018-10-15

**Authors:** Belinda De Simone, Luca Ansaloni, Micheal Denis Kelly, Federico Coccolini, Massimo Sartelli, Salomone Di Saverio, Michele Pisano, Gianfranco Cervellin, Gianluca Baiocchi, Fausto Catena

**Affiliations:** 1 Department of Emergency and Trauma Surgery, University Hospital of Parma, Parma, 43100, Italy; 2 Department of General and Emergency Surgery, Papa XXIII Hospital, Bergamo, 24121, Italy; 3 Acute Surgical Unit, Canberra Hospital, Canberra, ACT, 2601, Australia; 4 Department of Emergency and General Surgery, Macerata's Hospital, Macerata, 62100, Italy; 5 Department of General Surgery, Maggiore Hospital of Bologna, Bologna, 40121, Italy; 6 Department of Emergency Medicine, University Hospital of Parma, Parma, 43100, Italy; 7 Department of General Surgery, University Hospital of Brescia, Brescia, 25100, Italy

**Keywords:** Congress Impact Factor, HIndex, Educational Program, Scientific Quality, Academic Curriculum

## Abstract

Many scientific congresses and conferences are held every year around the world. The aim of the World Society of Emergency Surgeons.it (WSES) and Academy of Emergency Medicine and Care (AcEMC) was to develop a simple mathematical parameter as an indicator of academic quality and scientific validity of a congress. In this opinion article, a new metric, the Congress Impact Factor (IFc), is proposed taking into consideration the widely used Impact Factor as an indicator of journals’ prestige and using H-index analysis.

The IFc is derived from the mathematical ratio between the mean H-index of invited lecturers normalized for lecture topic and number of lectures in the conference. In case of multiple sessions, the mean of all IFc is calculated along with its standard deviation.  We conclude that the IFc can be a useful measure for evaluating and comparing congress prestige, and may also represent a potentially useful parameter for improving academic curriculum and helping participants to choose the more prestigious meetings for their education.

## Introduction

Many scientific congresses, meetings and conferences are organized each year around the world. Each congress can be promoted by a scientific society, which supports and organizes scientific sessions choosing topics and inviting national and international scientists as discussants, speakers or chairs. The choice of attending a specific congress is largely based on personal preferences, scientific area of interest and/or research, or simply as a desire to investigate, update and discuss topics of scientific relevance within the scientific community. The identification of the most useful and prestigious congresses and conferences organized by scientific societies is challenging, especially for young doctors who have not yet garnered a sufficient level of expertise. The scientific impact of a congress can only be valuable when supported by a good scientific program; the lectures delivered by experts in the field are essential for analyzing and discussing different medical and surgical topics ^1^.

The journal Impact Factor (IF), originally conceived by Irving H. Sher and Garfield in the early 1960s, is a bibliometric parameter aimed to evaluate journals’ prestige. It is usually calculated by dividing the number of citations in the previous two years to the number of citable items published in the same period ^2^. Therefore, a journal IF is based on two elements: the numerator, which is the number of citations in the current year to items published by the journal in the previous two years; the denominator, which is the number of citable items published in the previous two years ^3, 4^. Information about citations is obtained from a database now maintained by Clarivate Analytics (formerly by the Institute for Scientific Information). The list of journals’ IF is then published in the InCites Journal Citation reports, which is hence a useful means for establishing the absolute and relative (i.e., within a specific scientific field) prestige of a journal. Notably, albeit originally conceived for evaluating journals’ prestige, the IF is occasionally used also for evaluating scientists according to the number of articles published in high-IF journals ^5– 7^.

Unlike the IF, the H-index is a different metric used to evaluate scientists’ prestige according to the number of citations https://scholar.googleblog.com/2012/04/google-scholar-metrics-for-publications.html ^8^. The H-index was suggested in 2005 by Jorge E. Hirsch as a tool for determining theoretical physicists' relative quality ^9^ and is sometimes called the Hirsch index or Hirsch number. The definition of the H-index is that a scholar with an index of x has published x papers each of which has been cited in other papers at least x times ^10^. Consequently it involves the number of publications and the number of citations for publication to evaluate the scientific activity of a researcher and not only the total number of citations or publications. The limit is that the H-index can only be properly used for comparing scientists working in the same field.

## The congress impact factor

The aim of this opinion article is to present a mathematical coefficient to assess the quality and the academic validity of a scientific congress, using the IF formula and H-index calculation to create a useful tool: the Congress Impact Factor (IFc).

### Calculation

We propose that the IFc is calculated using the following formula:

$$IFc=\frac{mean\;H\;index\;of\;lecturers\;normalized\;for\;lecture\;topic}{number\;of\;lectures\;on\;topic\;at\;congress}$$

Mean H-index of Lecturers normalized for lecture topic was calculated using Google Scholar by Publish or Perish Harzing.com. For example, to obtain a mean H index normalized for lecture topic by Publish or Perish program is very easy: you have to choose to send your query by Google Scholar, searching for the “Name” and “Surname” of the author; automatically you will obtain your H index. Then you narrow down the search field to lecture topic and obtain H-index normalized for topic, for that author. All results should be analyzed checking for the right scientist, excluding non-relevant ones.

Subsequently, the mean H-index of all lecturers at the congress, normalized for lecture topic, is calculated to obtain a mean H-index plus a standard deviation. This value is divided by the number of lectures given in the congress obtaining the IFc.

Then the mean of all standard deviations must be calculated.

***Considerations:***

- The Chair’s H-index is always excluded because they do not give lectures.
- Only invited lectures should be considered.
- Free paper presenters are excluded because their academic value is too unpredictable and variable as we do not know how much they can influence the literature in the future: will they be published? In which journal? Will they be cited? How many times will they be cited?
- In case of a multi-session congress, a mean of all sessions plus standard deviation should be calculated.

### Validation

***Methods.*** As an example, we calculated the IFc for the first day of the Open Abdomen International Consensus Conference held in Dublin on July 2016. This was a consensus conference on critical surgical abdomen that produced guidelines on indications and benefits of open abdomen in non-trauma patients, which were published in the World Journal of Emergency Surgery ^11^. There were no other published proceedings of this conference. To create a comparison, we performed the H-index calculation for the same lecturers normalized for a different topic, “acute” “leukemia”, where none of the lecturers had specific expertise. The following mesh-words were used by Publish or Perish to calculate the H-index for every lecturer and mean H index in the two different topics ( Table S1): "Name Surname" and "open abdomen" and for the other evaluation "Name Surname" and "acute leukemia”. The comparison was made by the Student's *t*-test Statistical analysis was performed using IBM SPSS Statistics 22. P<0.05 was considered significant.

***Results.*** Invited speakers attending the two sessions of first day were 14 international emergency and trauma surgeons with a specific expertise in the open abdomen field. Table S1 shows the results of the IFc calculation based on normalized H-index for topic. The mean normalized H-index for open abdomen was 13.57 (SD 8.033), and the IFc was 0.96. The mean normalized H-index for the same speakers with a topic outside their expertise (acute leukemia) is 1.85 (SD 1.80; Table S1). The IFc for this hypothetical congress was 0.13. The difference between normalized H-index calculated between these two topics was statistically significant (p=0.0001).

## Discussion

In evaluating the quality and quantity of publications, two major categories of bibliometric indicators are available: quantitative indicators that measure the research productivity of a researcher; performance indicators that evaluate the quality of publications ^12^. The H-index is one of many available bibliometric indicators and is the most popular one to evaluate the academic and scientific activity of a researcher ^6^. In 2005, physicist Jorge E. Hirsch developed this index as a process for quantifying the output of an individual researcher. Hirsch stated: “I propose the index H, defined as the number of papers with citation number ≤ h, as a useful index to calculate the scientific output of a researcher” ^9^.

The H-index can be very useful in conceiving the IFc as a parameter to assess scientific quality of countless congresses and conferences that are proposed every year by scientific societies. The scientific impact of a congress is measured by a scientific program worthy of attention. We propose this simple indicator to measure quality of a Congress program based on the quality of its invited lecturers. The IFc involves the H-index combining it with IF calculation principles “to dilute” citation parameter with number of published articles. For IFc “the dilution” is performed with the number of lectures planned at the congress. We use the scientific potential given by the H-index of Lecturers invited/called to participate at the congress, normalized for the specific topic, avoiding the possibility that a highly cited scientist could give a lecture on a field outside their expertise, decreasing their educational effect. Dividing normalized H-index with the number of lectures, we can achieve a real-time picture of the quality of the educational meeting with clear evidence of the congress’s scientific impact. Only a limited number of good quality lectures is the source of a significant IFc with effective education of congress participants.

IFc is based on the H-index, which is actually considerated an indicator of scientific quality, and the IF philosophy. Currently they are both used to evaluate the strength of a scientist and of a scientific journal.

In conceiving the IFc, we reviewed the literature about the H index and we are aware of the criticism reported about the H index as a realistic indicator of the quality of work of one author.

The initial idea of Hirsch was to discriminate the investigators who are persistently productive from those who experienced an isolated auspicious moment in their scientific life. By time, we realized that the H index assumes that researcher A, who published a study that was extensively cited, should deserve less respect than researcher B who publishes often and regularly ^13– 15^.

With the H-index it is impossible to compare the investigators during different stages of their careers (even assuming comparisons among those representing the same field, which is another ambiguous factor). There is a certain correlation between the age of an investigator and H-index. Clearly some articles will accumulate citations and this number will increase over the time since they were first published ^13– 15^.

Another issue contributing to H-index limitations is that many research groups have different regulations regarding authorship. It is assumed that a researcher's name will be added to the authorship list only after considerable contribution has been made to the published work. However, what occurs fairly often is that being a “middle man” on the listing does not necessary reflect the significant contribution and, worth to be emphasized, the H-index does not differentiate between article authors who hold the most valuable first and last authorship position and those wherein the author's name appears as one among many other listed authors ^13– 15^.

Furthermore, the H-index does not discriminate self-citations and friendly cross-citations: it is not difficult to predict that even the investigators who are poorly cited by others but who publish prodigiously, citing mostly themselves or cited by friends-colleagues, will easily increase their H-index ^13– 15^.

Being aware of all these unsolved issues and considering all the indicators lately proposed to meet the need of more realistic and precise measures of the scientific activity of an author, the H-index remains the most used bibliometric indicator and we decided to use it to calculate the IFc.

IFc is able to describe the scientific expertise of lecturers on a specific topic with a quantitative evaluation of the quality of the meeting. For validation, we calculated the IFc for the WSES Consensus Conference on Open Abdomen: this was a high level meeting on a particular topic (open abdomen) where international experts are invited. The results of the validation of IFc suggest that the IFc can be an effective qualitative/quantitative metric for assessing congresses.

One limitation of IFc is that it would be difficult to calculate IFc in cases of a very large and heterogeneous congresses (e.g. American College of Surgeons). This is because many different symposiums have to be evaluated but the final IFc could be the mean of all these different IFcs using standard deviation to analyze the dispersion.

To the best of our knowledge there is nothing like the formal IF for conferences. In the past, conference proceedings publications were used to rate “lower quality” as compared to other “higher quality” congresses, especially if articles were published in peer reviewed international journals that are included in Thomson Reuters Journal Citation Reports http://wokinfo.com/products_tools/multidisciplinary/webofscience/cpci/. However with this system it is possible to have a retrospective and quite delayed information which is not so useful for choosing a congress prospectively. In other cases, conference proceedings were ranked in Thomson Reuters using “Conference Proceeding Citation Index”, but this is not comparable with an IF, and in this case you have retrospective and inaccurate information (evaluation of the congress is done a posteriori and without taking in consideration the lecturers). There is also the CORE Conference/Journal Ranking http://www.scimagojr.com/journalsearch.php?q=conference&tip=jou; http://arnetminer.org/page/conference-rank/html/All-in-one.htm, but again it is not a parameter based on strong indicators. There are other sources that could prove useful as an estimate of conference quality. Google Scholar lists top venues mixing journals and conferences in their listings. They list H-index of the venue instead of an IF, but again this is a misleading information (a high H-index venue can organize a Congress with low H-index lecturers).

Choosing the best Congress to attend can be difficult, and especially so for young attendees. Residents, scientific researchers and students require an ideal metric system to use as an indicator of scientific quality of a congress, so they can have the possibility to join congresses with high scientific impact and build on a competitive academic curriculum.

We believe that the IFc is an effective evaluation tool for a scientific meeting and it can become a valid instrument in the selection of the most appropriate congresses to update our knowledge in a specific field of research. This can contribute to develop a competitive academic curriculum vitae, i.e. reporting in the curriculum vitae the different conferences attended with their respective IFc.

## Conclusions

Bibliometric indicators are essential to evaluate scientific activity both of a researcher and an institution, or a journal.

Many congresses are organized and held every year and analysis of the congress programs shows that not all have a high scientific quality, despite being sponsored by international scientific societies and biomedical companies. In addition fees are requested to participate, and consequently it is very important to attend the best meetings that can improve one’s knowledge of a specific topic. It is important to be able to have a measurement of the quality of any given conference. We propose the IFc as the mathematical ratio between the mean H-index of invited lecturers normalized for lecture topic and the number of lectures at the conference. We believe that the IFc can be a useful metric system to assess the scientific validity of a congress, helping attendees to choose the best quality meeting to attend.

## Data availability

All data underlying the results are available as part of the article and no additional source data are required.

## Supplementary material

Table S1: Example of IFc calculation for Open Abdomen Congress 2016, in comparison with IFc for hypothetic Acute Leukemia Congress with the same authors.
