# Supplementary material for: The Congress Impact Factor: A proposal from board members of the World Society of Emergency Surgeons.it (WSES) and Academy of Emergency Medicine and Care (AcEMC)
Source: F1000Res. 2018 Oct 15;7:1185. Originally published 2018 Aug 3. [Version 2] doi: 10.12688/f1000research.15429.2 (PMC6208567; doi:10.12688/f1000research.15429.2)
Supplement: Supplementary file 1 [file f1000research-7-18107-s0000.tgz › 9f89af58-20d2-46c2-a919-6c88f5d94006.docx]

**Table S1: Example of Congress Impact Factor (IFc) calculation for the first day of the Open Abdomen Congress 2016, in comparison with IFc for hypothetical Acute Leukemia Congress with the same lecturers.**

| **Speakers** | **H Index** | **Normalized H Index for Open Abdomen** | **Normalized H Index for Acute Leukemia** |
| --- | --- | --- | --- |
| Marc A. De Moya | 16 | 5 | 1 |
| Walter Biffl | 74 | 15 | 3 |
| Ari Leppaniemi | 45 | 20 | 0 |
| Marja Boermeester | 52 | 17 | 1 |
| Andrew Kirkpatrick | 58 | 19 | 3 |
| Andrew B. Peitzman | 57 | 14 | 4 |
| Bruno Monteiro Tavares Pereira | 8 | 1 | 2 |
| Luca Ansaloni | 37 | 12 | 1 |
| Ernest E.Moore | 124 | 34 | 6 |
| Michael Sugrue | 46 | 16 | 0 |
| Jeffry L Kashuk | 22 | 4 | 1 |
| Ronald Maier | 74 | 16 | 4 |
| Yoram Kluger | 34 | 11 | 0 |
| Boris Sakakushev | 11 | 6 | 0 |
| Mean and SD | 47 (29.65) | 13.57 (8.03) | 1.85 (1.80) |
| **IFc of congress** |  | **13.57/14 = 0.96** | **1.85/14 = 0.13** |
