# Supplementary material for: The Congress Impact Factor: A proposal from board members of the World Society of Emergency Surgeons.it (WSES) and Academy of Emergency Medicine and Care (AcEMC)
Source: F1000Res. 2018 Oct 15;7:1185. Originally published 2018 Aug 3. [Version 2] doi: 10.12688/f1000research.15429.2 (PMC6208567; doi:10.12688/f1000research.15429.2)
Supplement: Peer review history

# Peer review: Referee response for version 2

**DOI:** 10.5256/f1000research.18107.r39464

**Published:** 2018-10-30

**Authors:** Francesco Azzaroli

**Affiliations:** 1 Department of Medical and Surgical Sciences (DIMEC), University of Bologna, Bologna, Italy

I have read the revised version of the manuscript and I find it now ready for approval.

I have read this submission. I believe that I have an appropriate level of expertise to confirm that it is of an acceptable scientific standard.

# Peer review: Referee response for version 1

**DOI:** 10.5256/f1000research.16814.r37590

**Published:** 2018-09-17

**Authors:** Luca Luzzi

**Affiliations:** 1 Thoracic Surgery Unit, Department of Medicine, surgery and neuroscience, Siena University Hospital, Siena, Italy

The paper from Ansaloni and co writers, touch on some crucial aspects of scientific divulgation. The first is about the quality of teaching: especially for young surgeons or others specialists the meetings are probably the first and most important instruments of updating. However, meanwhile some international meetings represents the masterpiece in their fields, many others give a less impact or appear redundant with a lower quality. Having a standardized method to rank the meetings you can address young to achieve the best quality information avoiding waste of money and time. On the other hand a sort of scientific meeting ranking could help company to address their investments to the best offers of scientific training. Only a final consideration to take in account, the meeting ranking should consider that also local up to date meetings have still a value to improve the medical or scientific culture in the periphery because more accessible respect an international one. Could be reasonable to cure this aspect with a different standardised event classification, for example; international meetings, nationals, inter-study groups or up to date.

 In my opinion the paper is for sure worthy of publication and then open for discussion in the scientific arena.

I have read this submission. I believe that I have an appropriate level of expertise to confirm that it is of an acceptable scientific standard.

# Response

**Published:** 2018-10-06

**Authors:** belinda de simone

**Affiliations:** University Hospital of Parma, France

Dear Colleague,

Thank you for your opinion and suggestions. You have hit our aim in the proposition of the IFc as a tool to evaluate the scientific impact both of an international congress and of a meeting organized with the intent to update knowledge about a specific research's field. By the IFc, we can select the best congress/meeting to upgrade our academic curriculum.

# Peer review: Referee response for version 1

**DOI:** 10.5256/f1000research.16814.r36778

**Published:** 2018-08-30

**Authors:** Francesco Azzaroli

**Affiliations:** 1 Department of Medical and Surgical Sciences (DIMEC), University of Bologna, Bologna, Italy

I reviewed with interest the paper entitled “The Congress Impact Factor: a proposal….”and, as far as my knowledge goes, this is the first time that a metric evaluation of a medical congress is proposed.

 The authors propose to measure an impact factor based on the mean H-index of invited lecturers normalized for lecture topic (i.e. the H-index of an author limited to the topic of the invited lecture) related to the number of invited lectures.

 Obviously, this metric has several limitations that come both from the intrinsic original defects of the H-index and from the complexity of evaluating the quality of a conference and of the speakers.

 In fact, the H-index reflects only the number of papers that have received a certain number of citations and does not include any information about the real contribution of that author to the manuscript nor the number of self citations. Furthermore, it tends to increase with time with increasing number of citations even though that author is no more productive.

 Because of these limitations several attempts have been made to improve the H-index trying to take into account the contribution of that author to the paper or the period of activity of the researcher adjusting for the number of years since the first publication. Nonetheless, there still is no perfect index to measure the quality and quantity of research which may be affected by so many factors ^1^^-^^4^. In fact, some researchers that have deeply impacted the world of science do not have impressive H-index ^2^, ^3^.

 Dealing with the world of medicine there is another point to consider that is practical expertise. The professionalism of a physician is not represented by the H-index. We all know that being scientifically very productive does not always correspond to being an “hands on” expert and to measure the practical expertise is an even more challenging task. The implementation of such an index could significantly impact the choice of speakers and may leave out non productive “hands on” experts.

 Finally, the metric may be affected by the number of speakers; i.e. a small conference may see its H-index rise if just a few authors with high H-index are invited. In such a case, the median with the range may better reflect the overall composition of invited speakers.

 Despite all these observations, I believe this paper deserves publication in order to start a serious discussion about scientific conferences. However, I believe the road to develop an acceptable measure of the quality of a conference is still long and rough.

 Coming specifically to the paper I have the following comments:

- Page 4, last paragraph before the discussion section: it should not be “between these two congresses…” but “…topics…”
- The discussion section should be partially rewritten taking into account the comments I made above and the fact that the H-index is not so robust. The authors should acknowledge the limitations of the metric and the possible drawbacks.
- In the last paragraph of the discussion the authors state that the conference impact factor can become a valid instrument of education to develop a competitive academic curriculum vitae. I disagree with this concept since participating to a conference does not necessarily correspond to an improvement of the professional knowledge. In this view the CME program is more close to this concept than the IF of a conference that does not measure learning. I would erase this sentence limiting the conclusion to the fact that the IFc may represent the first step in developing a simple tool to evaluate scientific conferences.

I have read this submission. I believe that I have an appropriate level of expertise to confirm that it is of an acceptable scientific standard, however I have significant reservations, as outlined above.

## References

[1] BucurOAlmasanAZubarevRFriedmanMNicolsonGLSumazinPLeabuMNikolajczykBSAvramDKunejTCalinGAGodwinAKAdamiHOZaphiropoulosPGRichardsonDRSchmitt-UlmsGWesterbladHKeniryMGrauGECarbonettoSStanRVPopa-WagnerATakharKBaronBWGalardyPJYangFDataDFadareOYeoKJGabreanuGRAndreiSSoareGRNelsonMALiehnEA: An updated h-index measures both the primary and total scientific output of a researcher. *Discoveries (Craiova)*.3(3) : 10.15190/d.2015.42 10.15190/d.2015.42 26504901PMC4617786

[2] MasicIBegicE: Scientometric Dilemma: Is H-index Adequate for Scientific Validity of Academic's Work?. *Acta Informatica Medica*.2016;24(4) : 10.5455/aim.2016.24.228-232 10.5455/aim.2016.24.228-232 PMC503798027708482

[3] KreinerG: The Slavery of the h-index-Measuring the Unmeasurable. *Front Hum Neurosci*.2016;10: 10.3389/fnhum.2016.00556 556 10.3389/fnhum.2016.00556 27853429PMC5089989

[4] AhangarHSiamianHYaminfiroozM: Evaluation of the Scientific Outputs of Researchers with Similar H Index: a Critical Approach. *Acta Informatica Medica*.2014;22(4) : 10.5455/aim.2014.22.255-258 10.5455/aim.2014.22.255-258 PMC421642925395728

# Response

**Published:** 2018-10-06

**Authors:** belinda de simone

**Affiliations:** University Hospital of Parma, France

Dear Professor Azzaroli,

Thank you to have review our opinion paper. 

We agree with you in highlighting limitations of the H index. We followed your suggestions and modified the manuscript considering all the issues you reported and upgrading the references, as you can check in the updated version of the paper.

Aware of all the criticism existing in literature about the real value of the H index but at the present there is no other indicator that can substitute it.
